# Crystal structure and functional characterization of an Asp49 phospholipase A_2_ from the bushmaster (*Lachesis muta*)

**DOI:** 10.1107/S2053230X26002736

**Published:** 2026-04-07

**Authors:** Noelia Erika Neyra Chama, Frey Francisco Romero Vargas, Eloy Condori Mamani, Jhon Antoni Vargas, Adriano Alves Furtado, Humberto D’Muniz Pereira, Ronald Demetrio Navarro Oviedo, Richard Charles Garratt, José Luis Javier Vega Ramírez, Diego Antonio Leonardo

**Affiliations:** a Laboratorio de Física Medica, Escuela Profesional de Física Universidad Nacional de San Agustin de Arequipa Avenida Independencia s/n Arequipa Peru; b Laboratorio de Bioquímica y Biología Molecular, Facultad de Ciencias Biológicas Universidad Nacional de San Agustin de Arequipa Avenida Alcides Carrión s/n Arequipa Peru; c Instituto de Física de São Carlos Universidade de São Paulo Avenida João Dagnone 1100 13563-723São Carlos São Paulo Brazil; University of São Paulo, Brazil

**Keywords:** snake-venom phospholipase A_2_, *Lachesis muta*, protein oligomer

## Abstract

Snake-venom phospholipases A_2_ (PLA_2_s) are small, structurally conserved enzymes that contribute significantly to the pathophysiology of envenomation. Here, we report the purification and crystal structure of an Asp49-PLA_2_ isolated from the venom of *Lachesis muta*, a pit viper from the Peruvian Amazon. The enzyme was purified using ion-exchange and size-exclusion chromatography and exhibited phospholipase activity in a dose- and time-dependent egg-yolk degradation assay. Pure protein crystals were obtained in space group *P*6_2_22 and diffracted to 2.36 Å resolution, with two molecules in the asymmetric unit. The structure reveals the canonical fold of catalytically active group II PLA_2_s, with a bound Ca^2+^ ion and a MES molecule in the active site of one monomer. Seven disulfide bonds stabilize the structure, although one bridge typically associated with the β-hairpin is absent and is replaced by a salt bridge as in other viperid PLA_2_s. *PISA* analysis suggests a potential tetrameric assembly composed of two *AB* dimers generating an interface between two *A* subunits (*A*–*A*′). Electrostatic surface mapping reveals a notable positively charged channel at the *A*–*A*′ interface, like that seen for a basic PLA_2_ homodimer from *Crotalus durissus terrificus* in which the two active sites lie accessible to the membrane. This study presents the first structural and enzymatic analysis of an Asp49-PLA_2_ from *L. muta* and provides insights into its oligomeric assembly, electrostatic landscape and potential adaptations relevant to its role in venom toxicity.

## Abstract 2

The crystal structure of an Asp49 phospholipase A_2_ from *L. muta* is reported, revealing a tetrameric assembly, catalytic conservation and a polarized electrostatic surface potentially linked to membrane targeting.

## 1. Introduction

Snake venoms are intricate cocktails of bioactive compounds comprising inorganic and organic molecules with significant toxic and pharmacological potential (Diniz-Sousa *et al.*, 2023 ▸). These venoms have become invaluable models for developing therapeutic agents, as they exhibit a vast array of biological activities (Lyukmanova & Shenkarev, 2024 ▸). Each venom is species-specific, and even within a species intraspecific variability is common, influenced by factors such as geographical location, age and diet (Sousa *et al.*, 2022 ▸). In Peru, *Lachesis muta*, also known as ‘shushupe’ or ‘bushmaster’, is a large pit viper that inhabits the Peruvian jungle and is considered to be one of the most dangerous snakes due to the potency of its venom and its high incidence in envenomation (Yarleque-Chocas *et al.*, 2023 ▸). The venom of *L. muta* is a complex blend of toxins with distinct molecular weights, isoelectric points and biological activities, reflecting the adaptability and diversity of its toxic components (Cañas *et al.*, 2023 ▸; Colombini *et al.*, 2001 ▸; Stransky *et al.*, 2018 ▸).

The pathological effects of *L. muta* venom are diverse and severe, including proteolytic, hemorrhagic, coagulant, myotoxic, fibrinolytic, defibrinogenic and cytotoxic effects (Damico *et al.*, 2005 ▸, 2007 ▸; Stransky *et al.*, 2018 ▸). These effects result from the action of several major toxin families present in the venom, such as snake-venom metalloproteinases (SVMPs),serine proteases and phospholipases A_2_ (PLA_2_s). PLA_2_ enzymes (EC 3.1.1.4) play crucial roles in disrupting homeostatic processes and modulating cellular signaling pathways (Cerón *et al.*, 2020 ▸; Proleón *et al.*, 2022 ▸). These enzymes catalyze the hydrolysis of the sn-2 ester bond of glycerophospholipids, releasing lysophospholipids and free fatty acids such as arachidonic acid, a precursor of pro-inflammatory mediators (Six & Dennis, 2000 ▸; Castro-Amorim *et al.*, 2023 ▸). Their catalytic mechanism is dependent on a well conserved active site consisting of essential residues such as His48, Tyr52 and Asp49, the latter of which is responsible for calcium ion co­ordination (Six & Dennis, 2000 ▸; Castro-Amorim *et al.*, 2023 ▸).

In viperid venoms, PLA_2_s are classified into two main subtypes: Asp49-PLA_2_s and Lys49-PLA_2_s. Asp49-PLA_2_s have catalytic activity due to the presence of aspartic acid at position 49, which plays a key role in the enzymatic hydrolysis of phospholipids (Six & Dennis, 2000 ▸; Castro-Amorim *et al.*, 2023 ▸; Ullah & Masood, 2020 ▸; Suranse *et al.*, 2022 ▸; Leite *et al.*, 2004 ▸). In contrast, Lys49-PLA_2_s, which have a lysine residue at position 49, lack enzymatic activity but still exhibit potent myotoxic effects through distinct, noncatalytic mechanisms. Both isoforms play essential roles in the toxic effects of snakebite envenomation, with Asp49-PLA_2_s being linked to hydrolytic activity and membrane disruption, while Lys49-PLA_2_s interact directly with cellular membranes, promoting calcium influx and necrosis (Lomonte, 2023 ▸). Venom constituents such as secreted phospholipase A_2_ enzymes (svPLA_2_s) and proteases are the most abundant and relevant in viperid snakes, but there are immense variations between them (Castro-Amorim *et al.*, 2023 ▸). This variability underscores the complexity of venom composition and highlights the importance of understanding the structural and functional properties of each toxin. In the toxic components of *L. muta* venom, PLA_2_s stand out as some of the most active and abundant molecules (de Oliveira *et al.*, 2024 ▸). These enzymes not only play critical roles in the pathophysiology of envenomation but have also been identified as potential pharmacological targets for the development of specific antivenoms (de Oliveira *et al.*, 2024 ▸; Six & Dennis, 2000 ▸; Castro-Amorim *et al.*, 2023 ▸). The structural characterization of venom components is crucial for understanding their mechanisms of action. The present study originated from a broader effort to fractionate and isolate components from *L. muta* venom. Among the purified fractions, one protein was crystallized and its structure was solved, later identified as an Asp49-PLA_2_. This work describes its purification, structural characterization and functional confirmation.

## 2. Materials and methods

### 2.1. Protein purification

Initially, we were interested in identifying the components of the venom and identifying possible phospholipases. With this in mind, the venom from the primary gland of *L. muta* (Cenepa-Alto Marañón, Amazonas, Peru) was manually extracted, lyophilized and stored at 10°C. A total of 250 mg of lyophilized *L. muta* venom was dissolved in 5 ml 50 m*M* ammonium acetate buffer pH 5.0. The solution was centrifuged at 2000*g* for 20  min at room temperature and the insoluble pellet was discarded.

The supernatant was applied onto a CM Sephadex C-50 ion-exchange chromatography column (28 × 2.6 cm) previously equilibrated with the same buffer at room temperature. Unbound proteins were eluted isocratically over three column volumes (CV) and monitored at 280 nm. Bound proteins were eluted at a flow rate of 1 ml min^−1^ using a stepwise NaCl gradient from 0 to 1 *M* consisting of the following phases: a linear increase from 0 to 30% NaCl over 0.5 CV, followed by a 1.5 CV plateau at 30% NaCl; a second linear increase from 30 to 60% NaCl over 0.5 CV, followed by a 1.5 CV plateau at 60% NaCl; and a final linear increase from 60 to 100% NaCl over 0.5 CV. A large number of peaks were observed in both phases of the separation. The fractions which eluted between 81 and 101 ml (‘peak 3’) were pooled and concentrated by centrifugation at 800*g* using an Amicon Ultra (3 kDa molecular-weight cutoff) centrifugal filter device (Merck Millipore, Darmstadt, Germany) prior to size-exclusion chromatography (SEC). This fraction was of particular interest because it was subsequently shown to present PLA_2_ activity (see below). The concentrated sample was loaded onto a Superdex 200 16/60 column previously equilibrated with 50 m*M* ammonium acetate buffer pH 5.0. The sample was injected at a volume of 2 ml and eluted at a flow rate of 1 ml min^−1^ at room temperature. The protein concentration prior to injection was not precisely determined at this stage and was adjusted based on the volume concentration. Protein purity was assessed by 15% SDS–PAGE under reducing conditions and the desired concentration was achieved by centrifugation at 800*g* using an Amicon Ultra (3 kDa molecular-weight cutoff) centrifugal filter device (Merck Millipore, Darmstadt, Germany). Protein concentration was estimated by measuring the absorbance at 280 nm using a NanoDrop spectrophotometer. Concentrations were calculated directly from *A*_280_ values using the standard approximation that an absorbance of 1.0 at 280 nm corresponds to an approximately 1 mg ml^−1^ concentration for typical proteins. Samples were kept frozen at −80°C for future use.

### 2.2. Enzyme activity

The phospholipase A_2_ activity of the major peak obtained from size-exclusion chromatography was assessed *in vitro* to confirm its functional identity, using a protocol adapted from Marinetti (1965 ▸). Briefly, different concentrations of the purified protein were tested to evaluate the degradation of lecithin complexes present in egg yolk. The purified fraction was diluted in 0.9%(*v*/*v*) saline and applied onto 96-well flat-bottomed ELISA plates (Corning) to obtain final concentrations of between 1.9 and 25 µg ml^−1^ in a final volume of 100 µl. 100 µl 2%(*v*/*v*) egg-yolk emulsion in saline was then added and the plates were immediately incubated at 42°C with shaking. Absorbance was measured at 925 nm using a spectrophotometer (Thermo Scientific Multiskan SkyHigh Microplate). Saline plus egg emulsion without protein was used as a negative control. The assay lasted 30 min, with readings taken every 5 min to monitor degradation of the egg-yolk emulsion.

### 2.3. Crystallization, data collection and structure determination

The major peak from the SEC which had been shown to exhibit phospholipase activity was crystallized by the sitting-drop vapor-diffusion method using the SG1 screening kit from Molecular Dimensions. Crystallization trials were set up using an automated crystallization robot (Crystal Gryphon, Art Robbins Instruments) in Intelli-Plate 96-3 low-profile plates (Art Robbins Instruments). Drops consisted of 0.2 µl protein solution (8.0 mg ml^−1^) mixed with 0.2 µl reservoir solution and were equilibrated against 30 µl reservoir solution. Plates were sealed with ClearVue adhesive sealing film (Molecular Dimensions) and were incubated at 291 K. After seven days, crystals were observed in the drop suspended over reservoir solution consisting of 0.2 *M* sodium acetate tri­hydrate, 0.1 *M* MES pH 6.0, 20%(*w*/*v*) PEG 8000. The crystals were harvested and cryocooled (cryoprotected by supplementing the reservoir solution with 20% PEG 200) in liquid nitrogen for subsequent data collection. X-ray diffraction data were collected using a PILATUS 2M detector on the MANACÁ beamline of the Sirius synchrotron, Laboratório Nacional de Luz Síncrotron–Centro Nacional de Pesquisa em Energia e Materiais (LNLS–CNPEM), Campinas, Brazil. All datasets were indexed and integrated using the *autoPROC* software (Vonrhein *et al.*, 2018 ▸) and scaled with *AIMLESS* (Evans & Murshudov, 2013 ▸). The structure of the PLA_2_ enzyme was determined by molecular replacement with *MOLREP* (Vagin & Teplyakov, 2010 ▸) using an *AlphaFold* model generated from the AIY33771.1 (UniProtKB A0A0A1ENR3) sequence (Abramson *et al.*, 2024 ▸; Jumper *et al.*, 2021 ▸). Iterative rounds of refinement and model building were carried out using *phenix.refine* (Adams *et al.*, 2010 ▸) and *Coot* (Emsley & Cowtan, 2004 ▸). Data-collection and refinement statistics and PDB accession codes are summarized in Table 1 ▸.

### 2.4. Electrostatic potential calculation

The electrostatic potential of the *L. muta* Asp49-PLA_2_ and the CBd isoform from *Crotalus durissus terrificus* (PDB entry 6tmy; Nemecz *et al.*, 2020 ▸) was calculated using the *PBEQ Solver* module of *CHARMM-GUI* (Jo, Vargyas *et al.*, 2008 ▸; Jo, Kim *et al.*, 2008 ▸). For the *L. muta* PLA_2_, the structure was used with bound Ca^2+^ ions, whereas for the CBd isoform the sodium ions present in the structure were retained. The input structures were prepared with default parameters, including grid focusing (1.5 Å before and 1.0 Å after focusing) and a protein dielectric constant of 1.0. The system pH was adjusted to match the crystallization conditions of each protein: pH 6.0 for *L. muta* and pH 8.0 for *C. durissus terrificus*. Electrostatic potential and solvation energy were computed, and potential maps were generated with default dielectric constants (ɛ_p_ = 1.0 for the protein interior, ɛ_s_ = 80.0 for the solvent). The resulting electrostatic potential maps were exported in OpenDX format and were visualized in *PyMOL* v.3.1 using the recommended *CHARMM-GUI* visualization commands.

## 3. Results and discussion

### 3.1. Phospholipase A_2_ purification

The PLA_2_ described in this study was obtained during a broader fractionation of *L. muta* venom. In this initial step, multiple protein peaks were separated by ion-exchange chromatography, and each fraction was analyzed to identify pure components suitable for crystallization trials. Here, we focus only on the results obtained from one of these peaks.

**Figure 1:**
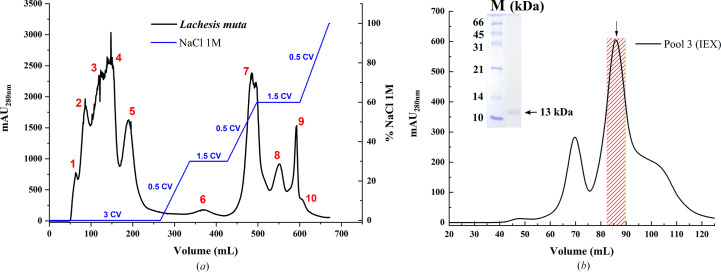
Purification of a protein fraction from *L. muta* venom. (*a*) Ion-exchange chromatography (IEX) profile on a CM Sephadex C-50 column. Several protein peaks were eluted both prior to and during the NaCl gradient (0–1 *M*), expressed in column volumes (CV) and indicated by the blue line. Peak 3 (81–101 ml) was selected for further purification as part of a broader fractionation aimed at isolating pure components for crystallization trials. (*b*) Size-exclusion chromatography (SEC) profile of peak 3 on a Superdex 200 16/60 column. The elution profile displays three peaks, with the most intense peak (highlighted as a shaded region and indicated by the arrow) containing a protein of approximately 13 kDa, as confirmed by 15% SDS–PAGE (lane M, molecular-weight marker, inset). The molecular-weight marker and the protein band correspond to different lanes from the same SDS–PAGE gel; the image was cropped and rearranged for clarity. This fraction was subsequently used for structural studies and for PLA_2_ activity measurements.

Purification was carried out in two sequential steps: ion-exchange chromatography (IEX) followed by size-exclusion chromatography (SEC). In the IEX performed on a CM Sephadex C-50 column, several proteins were eluted using the two phases (isocratic and a three-step linear gradient; Fig. 1 ▸*a*). The concentrated peak 3 fraction was subsequently subjected to SEC on a Superdex 200 16/60 column and showed three predominant peaks. SDS–PAGE analysis confirmed the presence of a single band with a molecular weight of approximately 13 kDa in the most intense peak (Fig. 1 ▸*b*). The molecular weight (∼13 kDa) observed for the purified protein falls within the range reported for other PLA_2_s characterized from snake venoms, which typically range between 13 and 15 kDa (Sousa *et al.*, 2022 ▸; Castro-Amorim *et al.*, 2023 ▸), suggesting that the protein could belong to this family. This two-step purification strategy proved to be highly effective in obtaining a homogeneous protein sample, which was subsequently used for structure determination. These results align with other studies on snake-venom PLA_2_, which report the effectiveness of combining IEX and SEC to isolate enzymatically active and structurally intact proteins (Sousa *et al.*, 2022 ▸; Chojnowski *et al.*, 2022 ▸).

### 3.2. Overall structure description

**Figure 2:**
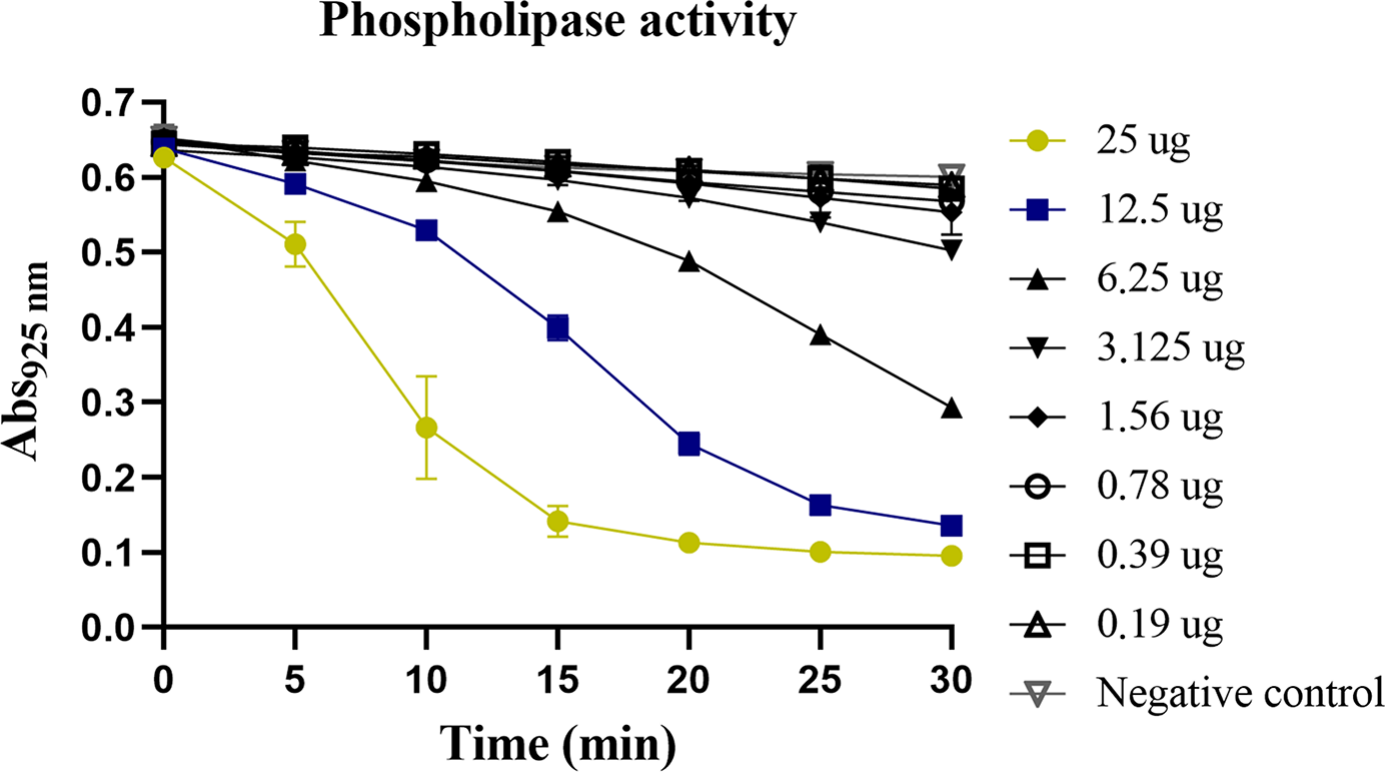
PLA_2_ activity of the purified protein from *L. muta*. The hydrolysis of egg-yolk phospholipids was monitored at 925 nm over 30 min using different protein concentrations. Higher protein concentrations (yellow and blue curves) showed a faster decrease in absorbance, consistent with phospholipid degradation. Saline plus egg-yolk emulsion without protein was used as a negative control.

The structure reported here was obtained from crystals of the purified protein described above (Supplementary Fig. S1). Initial sequence-independent molecular replacement was performed using the domain-search option of the *SIMBAD* program (Simpkin *et al.*, 2018 ▸), employing the *MoRDa* non­redundant domain database (Vagin & Lebedev, 2015 ▸). This approach uses a large collection of protein domains as search models in rotation-function screening, thereby allowing the identification of proteins whose identity is initially unknown, which was the present case. Using this domain-based search strategy, solutions consistent with PLA_2_-like domains were identified. Based on this structural indication, PLA_2_ sequences from *L. muta* were retrieved from public sequence databases (GenBank and UniProt). Among the available entries, the sequence AIY33771.1 (UniProtKB A0A0A1ENR3), corresponding to a PLA_2_ from *L. muta*, was selected due to its completeness and consistency with the expected molecular weight and fold. After removal of the signal peptide, an atomic model was generated using *AlphaFold* (Abramson *et al.*, 2024 ▸; Jumper *et al.*, 2021 ▸). This model was subsequently used as the search template for molecular replacement, as described in Section 2. After molecular replacement using the *AlphaFold*-derived model based on the sequence AIY33771.1 (UniProt A0A0A1ENR3), the resulting electron-density maps were carefully inspected throughout iterative cycles of model building and refinement. Residue identity was not assumed to follow any single database entry; instead, side-chain assignment at each position was evaluated directly against the 2*mF*_o_ − *F*_c_ and *mF*_o_ − *F*_c_ maps. In several positions, the electron density was clearly incompatible with the residue present in AIY33771.1 (for example, Gly22 instead of Ser), and alternative assignments were evaluated. In ambiguous regions, the residue identity was additionally assessed by comparison with conserved positions among closely related viperid Asp49-PLA_2_ sequences, including other *Lachesis* entries available in public databases (for example ADB77855.1 and C0HMB2.1). This structure-guided reassignment strategy ensured consistency between the experimental electron density, conserved structural features of the enzyme family and known sequence variation within viperid phospholipases. As a result, the final refined model differs from AIY33771.1 at a limited number of positions. The structure-based sequence shares 92.6% identity with AIY33771.1 (with nine substitutions), 97.5% with ADB77855.1 (another *L. muta* PLA_2_ entry, with three substitutions) and 98.4% with C0HMB2.1 (*L. acrochorda*, with two substitutions). This high level of conservation reflects the strong sequence similarity among viperid Asp49-PLA_2_ enzymes and is consistent with expected intraspecific and interspecific variation in snake-venom phospholipases. The deposited PDB model therefore represents the amino-acid sequence most consistent with the experimental electron density and conserved structural features of viperid Asp49-PLA_2_ enzymes, rather than a strict reproduction of any single database entry. Furthermore, during inspection of the difference maps, localized negative density was observed around several disulfide bonds and carboxylate side chains, consistent with site-specific radiation damage, a well documented phenomenon in disulfide-rich proteins. Although an explicit absorbed-dose calculation was not performed, these effects were localized and did not compromise the overall fold, disulfide connectivity or residue assignment, which remained well supported by the 2*mF*_o_ − *F*_c_ maps. Nevertheless, the precise redox state of individual disulfide bonds cannot be unambiguously inferred from the crystallographic data under these conditions. To confirm the functional identity suggested by the crystallographic analysis, the purified protein was assayed for phospholipase A_2_ activity using an egg-yolk degradation assay (Fig. 2 ▸). The enzyme displayed a concentration-dependent decrease in absorbance at 925 nm over time, consistent with the hydrolysis of phospholipids. These results verified that the crystallized protein retained catalytic function typical of Asp49-PLA_2_ enzymes.

**Figure 3:**
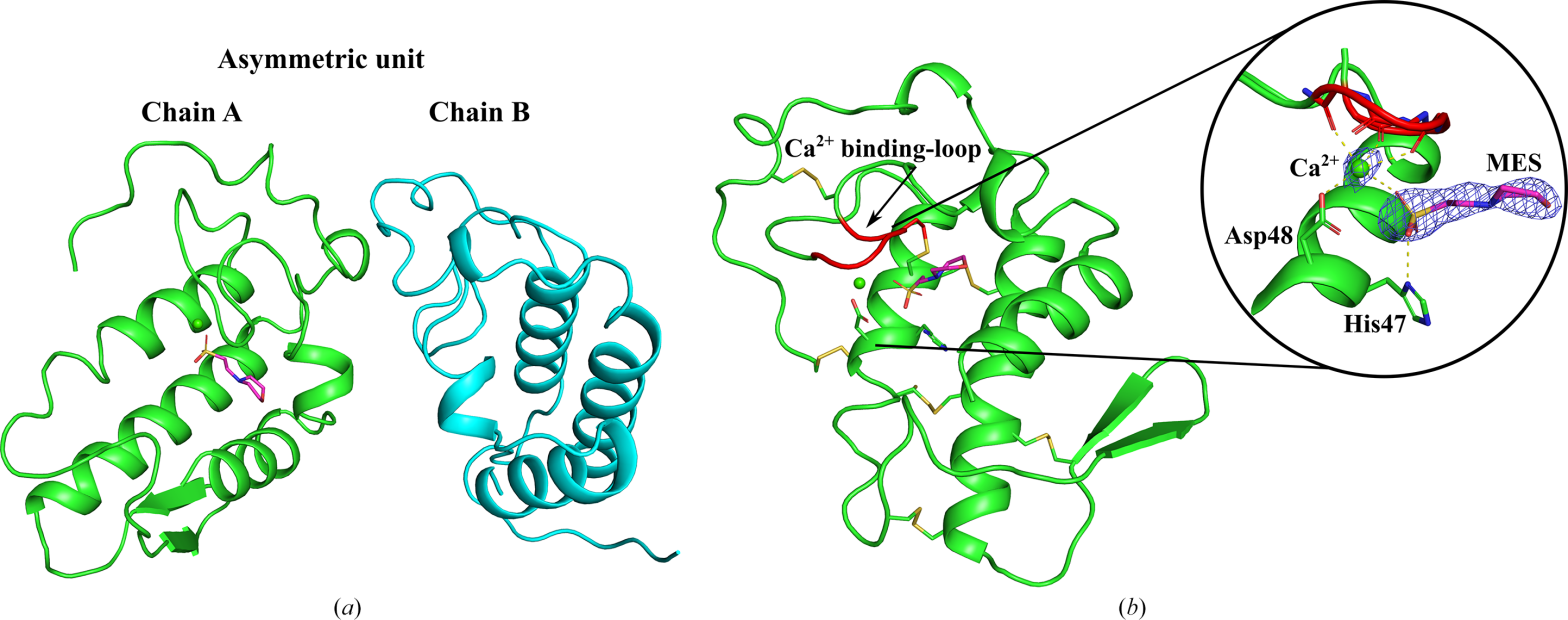
Crystal structure of the Asp49-PLA_2_ from *L. muta*. (*a*) Overall view of the asymmetric unit showing two independent molecules: chain *A* and chain *B*. (*b*) Close-up of chain *A* highlighting the Ca^2+^-binding loop and the active site. The inset displays the catalytic dyad formed by His47 and Asp48, the bound Ca^2+^ ion (green sphere) and a MES molecule from the crystallization condition, shown with its polder map (blue mesh) contoured at 2σ. The MES occupies a position within the active-site cleft and interacts with key residues, mimicking substrate recognition.

The structure of the phospholipase A_2_ (PLA_2_) isolated from *L. muta* was resolved at 2.36 Å (*R*_work_ = 24.30% and *R*_free_ = 26.37%) with two molecules in the asymmetric unit in space group *P*6_2_22 (Fig. 3 ▸*a*). All data-collection and refinement statistics are summarized in Table 1 ▸. Residue numbering throughout the manuscript follows the refined crystallographic model deposited in the PDB (PDB entry 9mle). Due to sequence-alignment differences and signal-peptide processing, the residue numbers in the present model may differ by one position from those reported in database annotations or the previous literature. Importantly, the functional classification of the enzyme as an Asp49-PLA_2_ is maintained according to the established PLA_2_ group numbering system (Schaloske & Dennis, 2006 ▸; Heinrikson *et al.*, 1977 ▸; Arni & Ward, 1996 ▸), which is based on structural alignment and conserved catalytic features rather than strict sequential numbering. Therefore, the catalytic aspartate responsible for Ca^2+^ coordination in our structure (Asp48) corresponds structurally to the canonical Asp49 residue described in the PLA_2_ literature.

Of the two subunits present in the asymmetric unit, only chain *A* displays bound ligands at the active site: a calcium ion (Ca^2+^) and a MES molecule. In contrast, chain *B* is empty of both. The presence of these ligands in chain *A* was confirmed by polder omit maps (Fig. 3 ▸*b*). The MES molecule, derived from the crystallization condition, occupies a position analogous to known substrate mimetics and interacts with the conserved catalytic dyad His47-Asp48, while also coordinating to the calcium ion. This active-site architecture supports the conserved catalytic mechanism typical of Asp49-PLA_2_ enzymes (Arni & Ward, 1996 ▸; Ward *et al.*, 2002 ▸; Lambeau & Gelb, 2008 ▸; Rouault *et al.*, 2006 ▸; Tonello & Rigoni, 2015 ▸).

Structural superposition of the two monomers reveals a high degree of conformational similarity, with a root-mean-square deviation (r.m.s.d.) of 0.4 Å across C^α^ atoms. However, local conformational differences are observed in the calcium-binding loop (Tyr27–Gly31) and the β-wing region comprising two short antiparallel β-strands and adjacent loops (which is disordered in the *B* chain). In other snake-venom PLA_2_, flexibility in the β-wing has been implicated in modulating catalytic efficiency and substrate interaction (Manjunatha Kini, 2003 ▸; Fernández *et al.*, 2013 ▸), although its specific role in *L. muta* remains to be determined.

**Figure 4:**
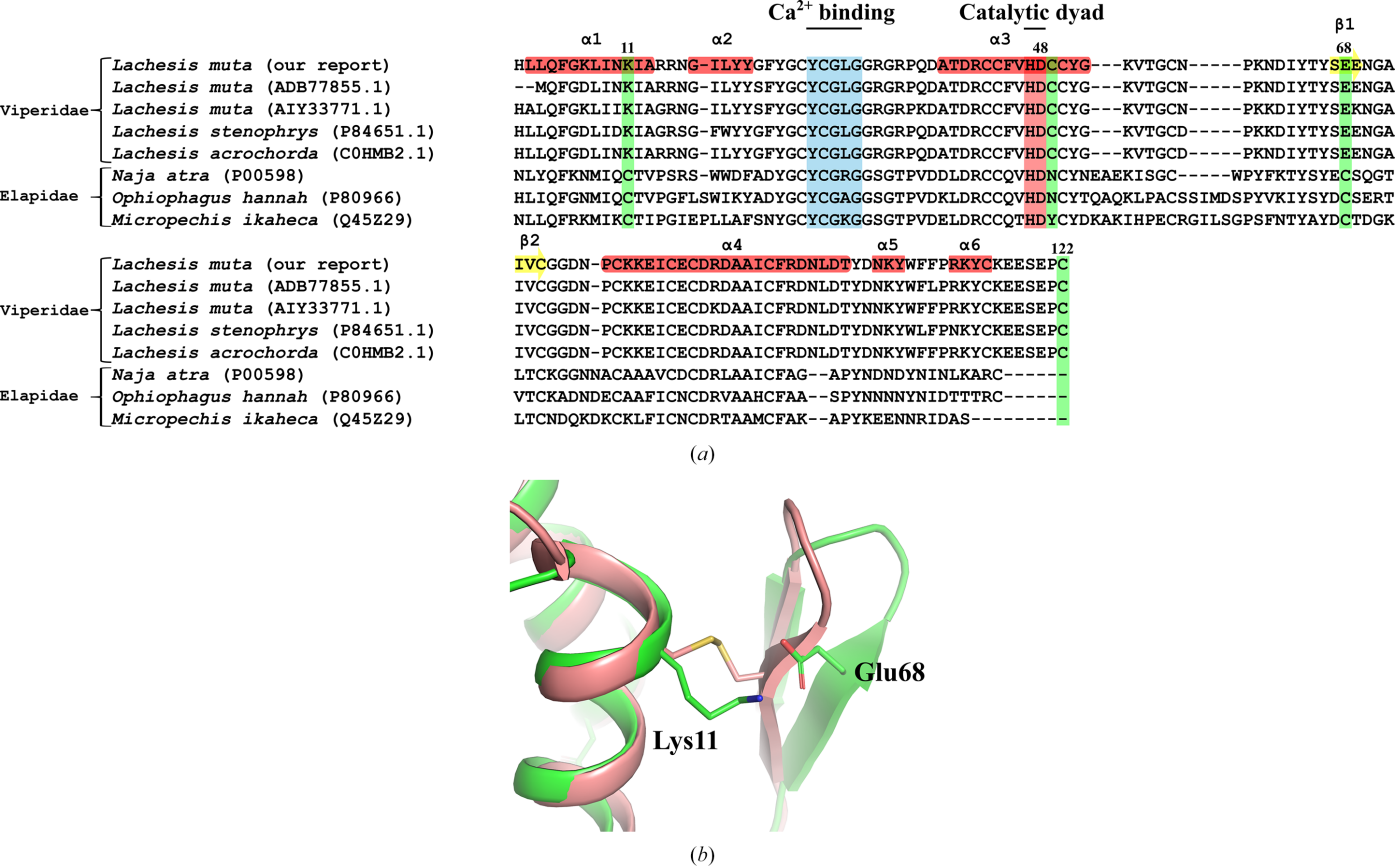
Sequence and structural comparison of the β-hairpin region in snake-venom PLA_2_s. (*a*) Multiple sequence alignment of PLA_2_ enzymes from group I (elapid) and group II (viperid) snakes. Residues involved in calcium binding and the catalytic dyad are highlighted. Cysteines required for the β-hairpin disulfide are conserved in elapid sequences but are absent in viperids, which possess a conserved C-terminal cysteine involved in an alternative disulfide bridge (residues 49 and 122). (*b*) Superposition of *L. muta* PLA_2_ (green) and that from an elapid species, *N. atra* (salmon; PDB entry 1poa), highlighting the β-hairpin and α1-helix regions. In *L. muta*, as in other viperid-derived PLA_2_s, the absence of the disulfide bridge connecting these elements is compensated by an electrostatic interaction between Lys11 and Glu68.

Snake-venom PLA_2_ enzymes have been structurally classified into two major groups: group I, predominantly found in elapid snakes, and group II, characteristic of viperid species, including *L. muta* (Dennis *et al.*, 2011 ▸; Salvador *et al.*, 2017 ▸). While both groups share a conserved three-dimensional fold and catalytic machinery, they differ primarily in the position of one of their seven disulfide bonds and in the presence of an extended C-terminal loop in group II enzymes (Dennis *et al.*, 2011 ▸; Salvador *et al.*, 2017 ▸). In the crystal structure of *L. muta* Asp49-PLA_2_, the disulfide bond typically observed between the first β-strand of the β-hairpin and the α1 helix, which is commonly conserved in group I PLA_2_s, is absent (Fig. 4 ▸*a*). Instead, in chain *A* we observe a potential electrostatic interaction between Lys11 and Glu68 that may contribute to stabilizing the β-hairpin/α1 region in the absence of this covalent linkage. Particularly, the corresponding region is less well defined in chain *B*, consistent with increased local flexibility and suggesting that this contact may be conformation-dependent rather than a constitutive feature (Fig. 4 ▸*b*). This correlates with a slight outward displacement of the β-hairpin relative to the center of mass of the protein, when compared with elapid PLA_2_s such as that from *Naja atra* (PDB entry 1poa; Scott *et al.*, 1990 ▸). Sequence alignment confirms that the cysteine required to form this disulfide bond is conserved in group I PLA_2_s (for example those from *Naja* and *Ophiophagus*) but absent in *Lachesis* and other viperids. Notably, group II PLA_2_s possess an extended C-terminal tail containing an additional disulfide bond not found in elapid sequences, which may contribute to structural stability despite rearrangement of the bonding network (Dennis *et al.*, 2011 ▸; Salvador *et al.*, 2017 ▸). This evolutionary shift in disulfide pattern may promote increased local flexibility in the β-wing region while maintaining global folding and enzymatic function. Such adaptations underscore the structural plasticity and functional diversification of snake-venom PLA_2_s (Borges *et al.*, 2023 ▸; Chioato *et al.*, 2007 ▸).

#### 3.2.1. Characteristics of the oligomeric state

**Figure 5:**
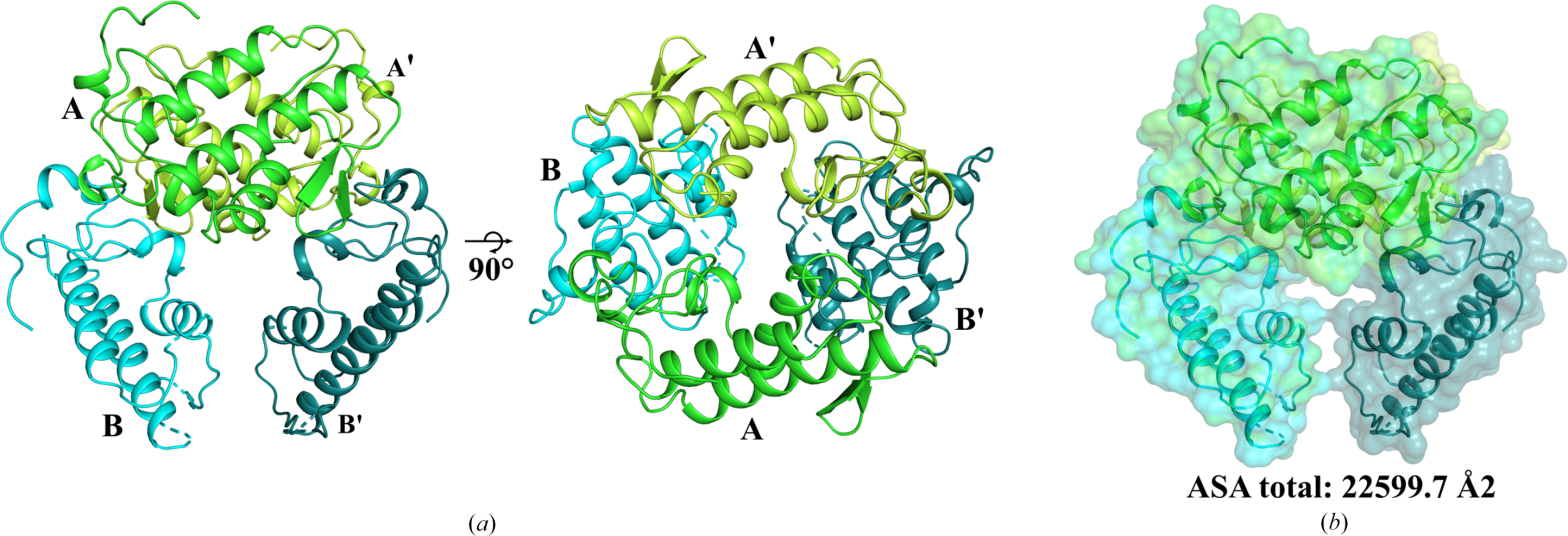
Tetrameric assembly of *L. muta* Asp49-PLA_2_ predicted by *PISA*. (*a*) Two views of the tetrameric quaternary structure comprising twofold symmetrically related dimers *A*–*B* and *A*′–*B*′. The arrangement reveals a symmetric, compact assembly. (*b*) Solvent-accessible surface representation of the tetramer showing the total accessible surface area (ASA) of 22 99.7 Å^2^.

It has been proposed that oligomerization enhances the functional versatility of PLA_2_ enzymes by promoting cooperative interactions and structural stability, particularly within venom-delivery systems (Damico *et al.*, 2005 ▸; de Oliveira *et al.*, 2024 ▸). Analysis of the quaternary structure of the Asp49-PLA_2_ from *L. muta* revealed a potential tetrameric assembly, as suggested by the *PISA* server (Krissinel & Henrick, 2007 ▸). This proposed assembly comprises two dimers (*A*–*B* and *A*′–*B*′) related by twofold symmetry, forming a tetramer with a total accessible surface area (ASA) of 22 599.7 Å^2^ (Fig. 5 ▸). The complex interfaces are stabilized predominantly by hydrophobic contacts and electrostatic interactions, which are consistent with previously reported oligomeric PLA_2_s (Matsui *et al.*, 2019 ▸; Gomes *et al.*, 2020 ▸; Marchi-Salvador *et al.*, 2008 ▸; Borges *et al.*, 2023 ▸).

**Figure 6:**
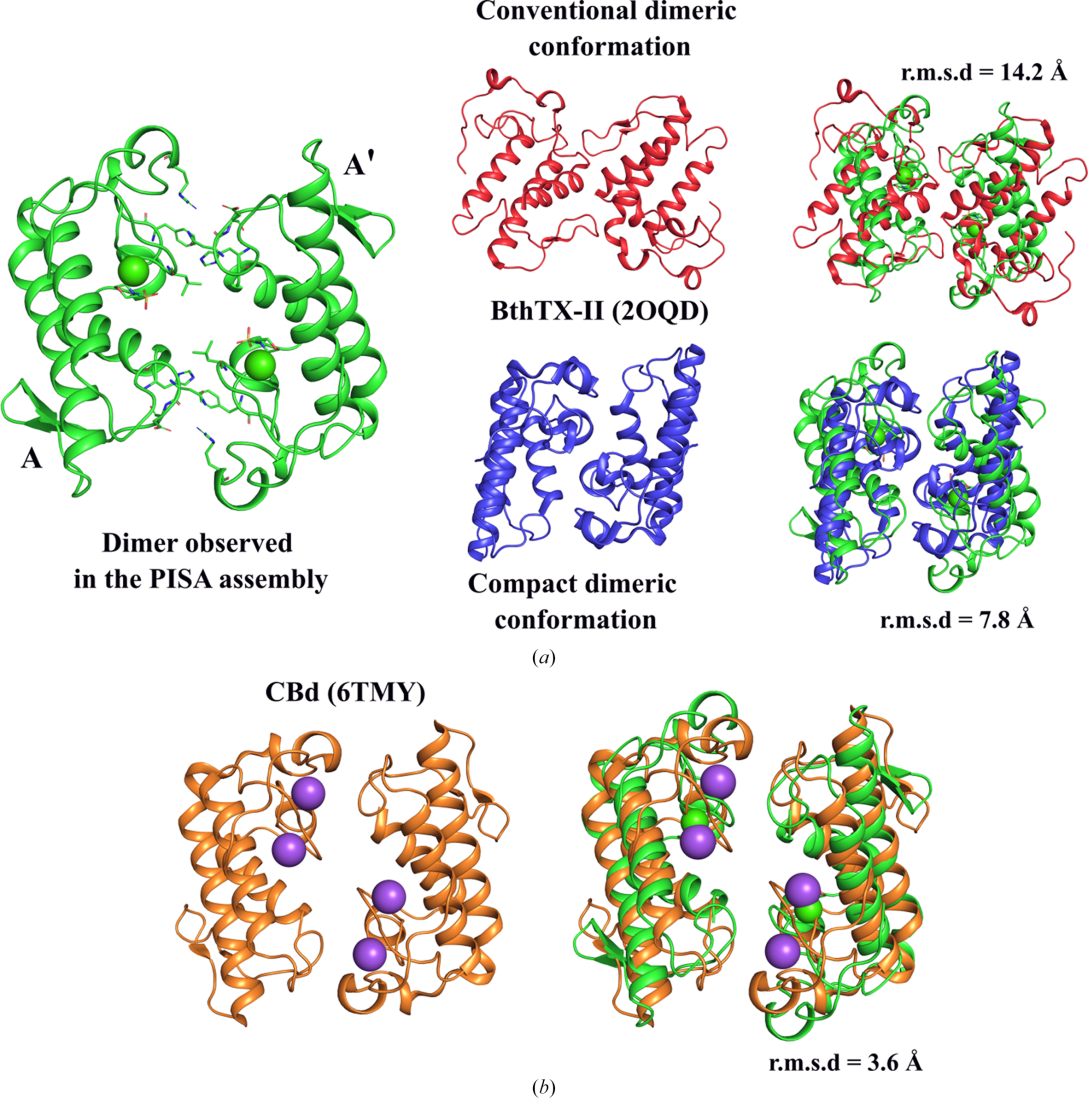
Structural comparison of dimeric conformations in snake-venom PLA_2_ enzymes. (*a*) Dimer formed by chains *A* and *A*′ in the *L. muta* Asp49-PLA_2_*PISA* assembly compared with the conventional and compact dimers of BthTX-II (PDB entry 2oqd; red and blue, respectively). Superpositions yielded r.m.s.d. values of 14.2 Å (conventional) and 7.8 Å (compact). (*b*) Comparison of the *L. muta* Asp49-PLA_2_ dimer (green) with the CBd isoform from *C. durissus terrificus* (PDB entrty 6tmy), showing an r.m.s.d. of 3.6 Å.

The *PISA* analysis predicts this tetramer to be the biologically relevant assembly, although several different dimeric assemblies have been reported for PLA_2_s in the past. It should be borne in mind that the asymmetric unit reported here has two chemically identical subunits which are crystallo­graphically (and structurally) distinct. While chain *A* displays well defined density for a bound Ca^2+^ ion and a MES molecule at the active site, chain *B* does not show interpretable electron density for these ligands under the present crystallization conditions. As calcium binding was not experimentally investigated in this study, the absence of Ca^2+^ in chain *B* should not be overinterpreted. Consequently, the dimeric interface observed between two *A* subunits (*A* and *A*′) in the tetramer is therefore of greatest interest and can be compared with those described in other viperid PLA_2_s. Two different arrangements have been described, and these have been named the ‘conventional’ and ‘alternative’ (or ‘compact’) dimerization modes, as seen in the crystal structure of BthTX-II (PDB entry 2oqd; Corrêa *et al.*, 2008 ▸; Marchi-Salvador *et al.*, 2008 ▸; Matsui *et al.*, 2019 ▸; Gomes *et al.*, 2020 ▸). To further explore this resemblance, we superimposed the compact dimer observed in the *L. mutaPISA* assembly (chains *A*–*A*′) with both dimers of BthTX-II, yielding r.m.s.d.s of 7.8 and 14.2 Å for the compact and conventional arrangements, respectively, clearly indicating that the assembly observed here does not correspond to either (Fig. 6 ▸*a*). On the other hand, a related arrangement has been observed in the case of one of the basic phospholipase A_2_ isoforms (CBd) from *C. durissus terrificus* (PDB entry 6tmy). In this case *PISA* predicts two potential tetramers, both of which include a pair of subunits which interact in a manner similar to that observed for the *A*–*A*′ pair described here, with an r.m.s.d. of 3.6 Å (Fig. 6 ▸*b*). Whether the full tetramer identified by the *PISA* analysis or this dimeric substructure represent physiologically relevant assemblies is an open question.

**Figure 7:**
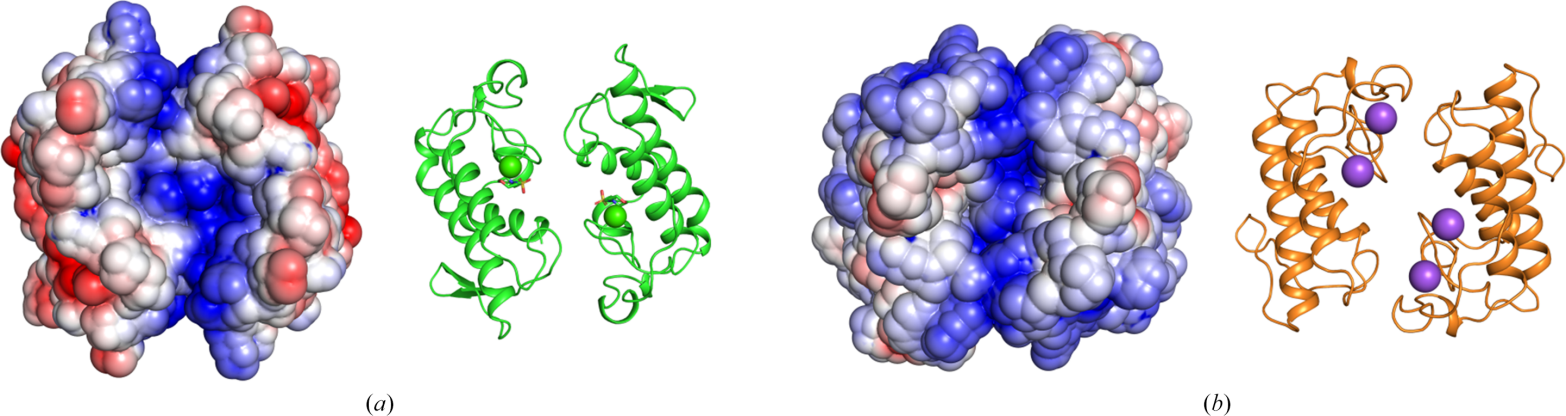
Electrostatic surface potentials of PLA_2_ dimeric conformations. (*a*) *A*–*A*′ dimer from the *L. muta* Asp49-PLA_2_*PISA* assembly. (*b*) Dimer from the CBd isoform of *C. durissus terrificus* (PDB entry 6tmy). Surface potentials are colored from −2.0 *kT*/*e* (red) to +2.0 *kT*/*e* (blue). Cartoon representations of the corresponding dimers are shown for reference.

The electrostatic surface potential of the *L. muta* Asp49-PLA_2_*A*–*A*′ dimer reveals an interesting charge distribution which is also observed in the dimer from *C. durissus terrificus* (PDB entry 6tmy). A channel of positive charge, which includes both active sites of the dimer, extends between the two subunits. As with other PLA_2_s (Castro-Amorim *et al.*, 2023 ▸; Teixeira *et al.*, 2011 ▸), regions of positive charge have been attributed to the interaction with negatively charged phospholipids in membranes. In the case of *L. muta* Asp49-PLA_2_ this region may be relevant both for initial membrane interaction and the subsequent attraction of the phospholipid substrate into the active site for hydrolytic cleavage, with the subsequent release of free fatty acid and lysophospholipid (Fig. 7 ▸).

Altogether, our findings provide the first structural and enzymatic characterization of an Asp49-PLA_2_ from *L. muta*. This contributes to the overall body of knowledge on these enzymes, whose complex list of biological activity has yet to be fully elucidated. The conserved catalytic features, distinct oligomeric organization and polarized electrostatic surface support a model in which quaternary structure and surface-charge distribution cooperate to enhance membrane targeting and enzymatic function. These results expand our understanding of structure–function relationships among viperid PLA_2_s and may inform future studies on venom evolution and inhibitor design.

## Supplementary Material

PDB reference: Asp49 phospholipase A_2_ isolated from *Lachesis muta*, 9mle

Supplementary Figure S1. DOI: 10.1107/S2053230X26002736/nq5002sup1.pdf

## Figures and Tables

**Table 1:** Data-collection and processing statistics for Asp49-PLA_2_

X-ray source	MANACÁ, Sirius
Detector	PILATUS 2M
*a*, *b*, *c* (Å)	98.19, 98.19, 120.52
α, β, γ (°)	90.00, 90.00, 120.00
Space group	*P*6_2_22
Resolution (Å)	49.17–2.36 (2.45–2.36)
Wavelength (Å)	0.9771
Multiplicity	37.1 (41.0)
*R*_p.i.m._ (all *I*^+^ and *I*^−^) (%)	1.6 (13.9)
CC_1/2_	0.997 (0.986)
Completeness (%)	99.9 (100.0)
Reflections	547256 (61749)
Unique reflections	14731 (1507)
〈*I*/σ(*I*)〉	29.5 (6.4)
Reflections used in refinement	14685
*R* (%)	23.69
*R*_free_ (%)	25.87
No. of protein atoms	1808
No. of water atoms	43
No. of ligand atoms	13
*B* factor (Å^2^)	50.84
Coordinate error (ML-based) (Å)	0.34
Phase error (°)	27.91
Ramachandran favored (%)	96.46
Ramachandran allowed (%)	3.10
All-atom clashscore	5.30
R.m.s.d., bond lengths (Å)	0.004
R.m.s.d., bond angles (°)	0.668
PDB code	9mle
