# Supplementary material for: Crystal structure and functional characterization of an Asp49 phospholipase A2 from the bushmaster (Lachesis muta)
Source: Acta Crystallogr F Struct Biol Commun. 2026 Apr 7;82(Pt 5):150–9. doi: 10.1107/S2053230X26002736 (PMC13133998; doi:10.1107/S2053230X26002736)
Supplement: Supplementary file 1 [file f-82-00150-sup1.pdf]

STRUCTURAL BIOLOGY
COMMUNICATIONS

Volume 82 (2026)

Supporting information for article:

Crystal structure and functional characterization of an Asp49 phospholipase A_2 from the bushmaster (*Lachesis muta*)

Noelia Erika Neyra Chama, Frey Francisco Romero Vargas, Eloy Condori Mamani, Jhon Antoni Vargas, Adriano Alves Furtado, Humberto D' Muniz Pereira, Ronald Demetrio Navarro Oviedo, Richard Charles Garratt, José Luis Javier Vega Ramírez and Diego Antonio Leonardo

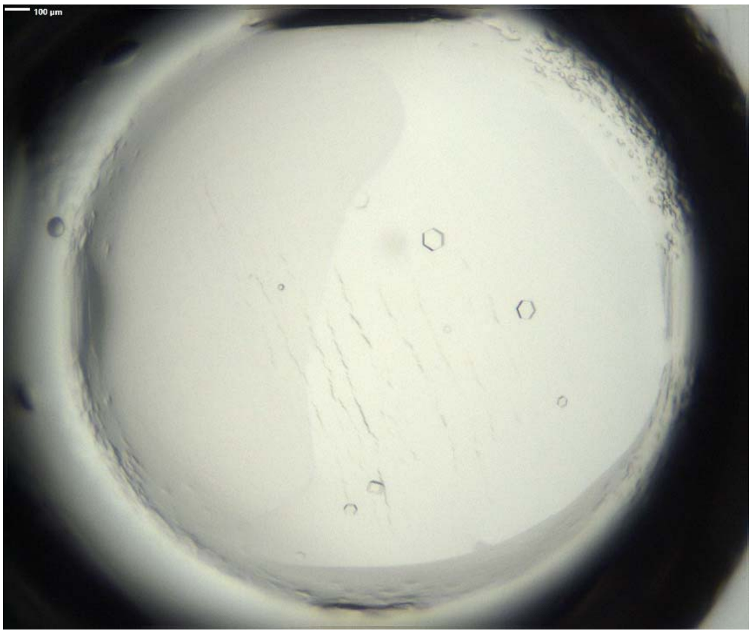

Figure S1 Representative crystal of Asp49 phospholipase A_2 from *Lachesis muta* obtained under the crystallization conditions described in the Methods section. The scale bar corresponds to 100 µm.
